# How Do Diabetes Models Measure Up? A Review of Diabetes Economic Models and ADA Guidelines

**DOI:** 10.36469/9831

**Published:** 2015-12-16

**Authors:** Lindsay Govan, Olivia Wu, Robert Lindsay, Andrew Briggs

**Affiliations:** 1 University of Glasgow, Glasgow, UK

**Keywords:** reporting guidelines, economic evaluation, computer simulation, lifetime outcomes, predictive model, diabetes

## Abstract

**Introduction:** Economic models and computer simulation models have been used for assessing short-term cost-effectiveness of interventions and modelling long-term outcomes and costs. Several guidelines and checklists have been published to improve the methods and reporting. This article presents an overview of published diabetes models with a focus on how well the models are described in relation to the considerations described by the American Diabetes Association (ADA) guidelines.

**Methods:** Relevant electronic databases and National Institute for Health and Care Excellence (NICE) guidelines were searched in December 2012. Studies were included in the review if they estimated lifetime outcomes for patients with type 1 or type 2 diabetes. Only unique models, and only the original papers were included in the review. If additional information was reported in subsequent or paired articles, then additional citations were included. References and forward citations of relevant articles, including the previous systematic reviews were searched using a similar method to pearl growing. Four principal areas were included in the ADA guidance reporting for models: transparency, validation, uncertainty, and diabetes specific criteria.

**Results:** A total of 19 models were included. Twelve models investigated type 2 diabetes, two developed type 1 models, two created separate models for type 1 and type 2, and three developed joint type 1 and type 2 models. Most models were developed in the United States, United Kingdom, Europe or Canada. Later models use data or methods from earlier models for development or validation. There are four main types of models: Markov-based cohort, Markov-based microsimulations, discrete-time microsimulations, and continuous time differential equations. All models were long-term diabetes models incorporating a wide range of compilations from various organ systems. In early diabetes modelling, before the ADA guidelines were published, most models did not include descriptions of all the diabetes specific components of the ADA guidelines but this improved significantly by 2004.

**Conclusion:** A clear, descriptive short summary of the model was often lacking. Descriptions of model validation and uncertainty were the most poorly reported of the four main areas, but there exist conferences focussing specifically on the issue of validation. Interdependence between the complications was the least well incorporated or reported of the diabetes-specific criterion.

## INTRODUCTION

### Rationale

Economic models have been used for assessing short-term cost-effectiveness of interventions as well as modelling long-term outcomes and lifetime costs in almost all disease areas. Decision makers often turn to computer simulation models to predict the effect of treatments in the longer term. These models can be used to address a variety of clinical outcomes and questions, and also assess new treatment strategies. Although clinical trials are excellent sources of information on effectiveness of treatments, they are only applicable to the population recruited, do not account for all characteristics of a population, and they tend to be short timescales.[1] This means that economics models are useful sources of information by providing estimates of the long-term effects and costs of new interventions.

Several guidelines and checklists have been published to improve the methods and reporting of economic models.[2-5] Philips et al[3] performed a systematic review to identify and summarise all guidelines that were available for assessing quality of decision-analytic models. They created a checklist for critically appraising decision-analytic models for Health Technology Assessment (HTA), based on the format by Sculpher et al.[4] The checklist suggests that models should clearly describe three major areas: structure such as clear statement of problems/objective, rationale for the structure, assumptions, comparisons, model types, time horizon, disease states and cycle lengths; the data used to develop and populate the model including how data was identified, modelled, and incorporated into the model, and assessing uncertainty; and consistency, meaning whether the model is performing the way it was intended to perform, both internally (by testing the mathematical logic of the model during development to fix errors) and externally (whether the results of the model are consistent with information contained in relevant primary research studies).

Other guidelines suggest that additional characteristics should be considered when assessing the quality of a model.[6,7] These include: clinical relevance, encompassing all important facets of the disease of interest; transparency, details of model structure and assumptions are provided with clear data sources; reproducibility, results of the model can be reproduced by an independent researcher; interpretability, results are clear and can be easily interpreted; and exploration of analytical ability and uncertainty such as methodological, structural, and data uncertainty, including heterogeneity and parameter uncertainty.

Recently a series of seven papers were published which updated the recommendations for best practices. The series provides a series of recommendations for each stage of model development, providing helpful suggestions on assessing the model for developers, reviewers, and those who report the results of models or use models to make decisions. The series provides best practice advice and recommendations on five main areas: model conceptualisation; implementation of specific types of model, including state-transition models (cohort or individual), discrete-event simulation, and dynamic transmission models; dealing with uncertainty and parameter estimation; validity and reporting models transparently.[8-14]

However, in addition to generic advice for developing, assessing and reporting of economic models, there are often disease specific considerations that should be included in the model development. One area where economic models have been used widely is diabetes. Diabetes is a chronic and complex disease with increased risks of cardiovascular complications in addition to diabetes specific complications. The prevalence and financial cost of diabetes is rising worldwide[15,16], meaning that understanding and accurately assessing the costs and effectiveness of healthcare delivery in diabetes is of clear importance to health services.

In 2004, the American Diabetes Association (ADA) convened a work group of diabetes modellers to create standardised guidelines that future modellers can use to ensure their models are accurate, useful and reliable.[17] Along with the main considerations for models, they work group all determined a list of diabetes-specific requirements for the models.

There are several diabetes models in existence that have been included in previous reviews. One review[18] focussed on assessing models used in drug treatment cost-effectiveness analysis and the treatment effects that are incorporated into the model. The authors focussed on the model's ability to incorporate the costs and benefits associated with different drug treatment alternatives. This authors found that most models share common data sources and modelling approaches, and differed in terms of interventions and complications evaluated. They conclude that models should be reported in more detail in order to make them more transparent by including assumptions, data and statistical methods used, and should aim to include a wider range of treatment outcomes relating to both the effects of diabetes and its complications, and also side effects of treatments investigated.

Tarride et al[19] provide an overview of models focussing on the details of the model itself such as type, structure, data sources, assumptions, validations, presentation or results, and treatment of uncertainty. Similar to Yi et al, the authors of this review found that most models used similar model types and data sources, but differed in the complications that were include in the model. The authors conclude that models could be enhanced if they were able to cope with both first- and second-order uncertainty.

Neither of the previous reviews assessed the quality of the reporting of the model in regards to the guidelines set out by ADA. This review identifies and critically appraises diabetes simulation prediction models used to calculate health economic outcomes. Specifically, this article presents an overview of published diabetes models with a focus on how well the models are described in relation to the considerations described by the ADA guidelines.

### METHODS

#### ADA Guidance

The American Diabetes Association are aware that decision makers are turning more to computer modelling in order to make decision on health care for those with diabetes. Models can be very powerful decision making tools if they are properly constructed, validated and applied. Therefore, a work group of diabetes modellers was convened to create standardised guidelines that future modellers can use to ensure their models are accurate, useful, reliable and reproducible, and to reassure model users of its quality.[17]

The workgroup determined there were three main considerations. First, models should be transparent by providing complete descriptions of the model's structure including inputs, equations, algorithms, assumptions and data sources. If the model is based on previously published model, changes and additions should be described in adequate detail. Second, authors should report the level to which a model was validated to allow readers to assess whether predictions made by the model are accurate, this can include internal validation (reproduces results of the studies that are used to develop the model) and external validation (reproduces results of studies that were not used to develop the model). Finally, methods of assessing uncertainty should be described. Five types are listed, including ignorance, known variability, statistical variability, Monte Carlo variability and uncertainty from the model design. Uncertainty should be address through sensitivity analysis, averaging over multiple simulations or seek results from multiple models to ensure accuracy of results.

In addition to these, the ADA workgroup also list seven diabetes-specific requirements for models: long-term time horizons to allow complications to occur at a sensible time but also include mortality as a competing risk; include complications for multiple organ systems and interdependence between complications; include treatment effects since they can affect a diverse range of outcomes; should include both life-expectancy and quality of life measures; select the perspective of the model carefully and explicitly state it in the analysis; be aware that there is a delay between onset and diagnosis; developers should be specific about the criteria used to diagnose and classify diabetes since the diagnostic criteria have changed over time. The final two requirements were not investigated in this review since we were interested in models predicting lifetime outcomes post-diagnosis.

#### Literature Review

The National Health Service Economic Evaluation database (NHS EED), Ovid, MEDLINE, and EMBASE were searched in December 2012 to identify possibly relevant articles. A combination of medical subject headings (MESH) and relevant keywords were used. Search terms were combined with Boolean operators OR and AND. These included terms for the disease area (diabet$, diabetes, diabetes mellitus), study type (analys$, evaluat$, model$), type of model (predict$, simulate$, lifetime, computer simulation), and incorporating health economic components (cost-effectiv$, cost-utility, life-years gained, quality-adjusted life-year, QALY, economic$, economic evaluation, cost$).

Studies were included in the review if they estimated lifetime outcomes for patients with type 1 or type 2 diabetes. All types of models were included. Studies were excluded if they were clinical studies, cost or cost-effectiveness studies only (i.e. not simulation studies), non-diabetes related, or if title identified the article as a screening or preventative model. Studies were further excluded if they only modelled one type of diabetes complication (e.g. retinopathy), or only a subgroup of patients (e.g. overweight people with diabetes). The search was not restricted by date or language.

Titles and abstracts were screened to identify relevant publications. Only unique models were included in the review. If multiple papers reported using a particular model, the original paper was included in the review. However, for models where additional information was reported in subsequent or paired articles, then the additional citations were also included. Further to the electronic database searching, NICE guidelines were also searched. References and forward citations of relevant articles, including the previous systematic reviews were also searched using a method similar to the pearl growing method.[20]

A data extraction form was created in Excel. In order to assess each models' reporting of the ADA guidelines the following details were collected: model aims/objectives; type of diabetes; model structure and simulation technique; data sources for patient data, costs, utilities, and methodologies; modelled complications/events; and outcomes and outputs from the model, such as life expectancy, QALY. These details were collected to assess model transparency, and to assess how the model incorporated the additional diabetes-specific considerations as suggested by the ADA guidelines.

### RESULTS

#### Search results

Figure 1 provides an overview of the number of studies identified, included, excluded and reasons for exclusion. In total 2389 citations were identified from electronic citation searching, of which 2341 were excluded based on title and abstract. Reference searching proved to be more efficient at identifying relevant articles due to that vast number of economic evaluations performed in diabetes research. A total of 97

citations were extracted for full text review from electronic citation searching, and an additional 52 articles identified from forward citation searching and reference searching. Models that included a screening component were excluded from this review. However, models that used or extended the post-diagnosis component of a screening model in order to create a new model[21, 22] were included.

After excluding articles based on the criteria described, a total of 28 articles were considered relevant to the review. This number does not represent the number of identified models but the number of articles that describe the models, including companion and paired articles which describe validation, methodology etc. After reviewing and grouping the articles, a total of 19 models were identified.

**Figure 1.** Flow Chart of Publications Selection and Exclusion from the Review

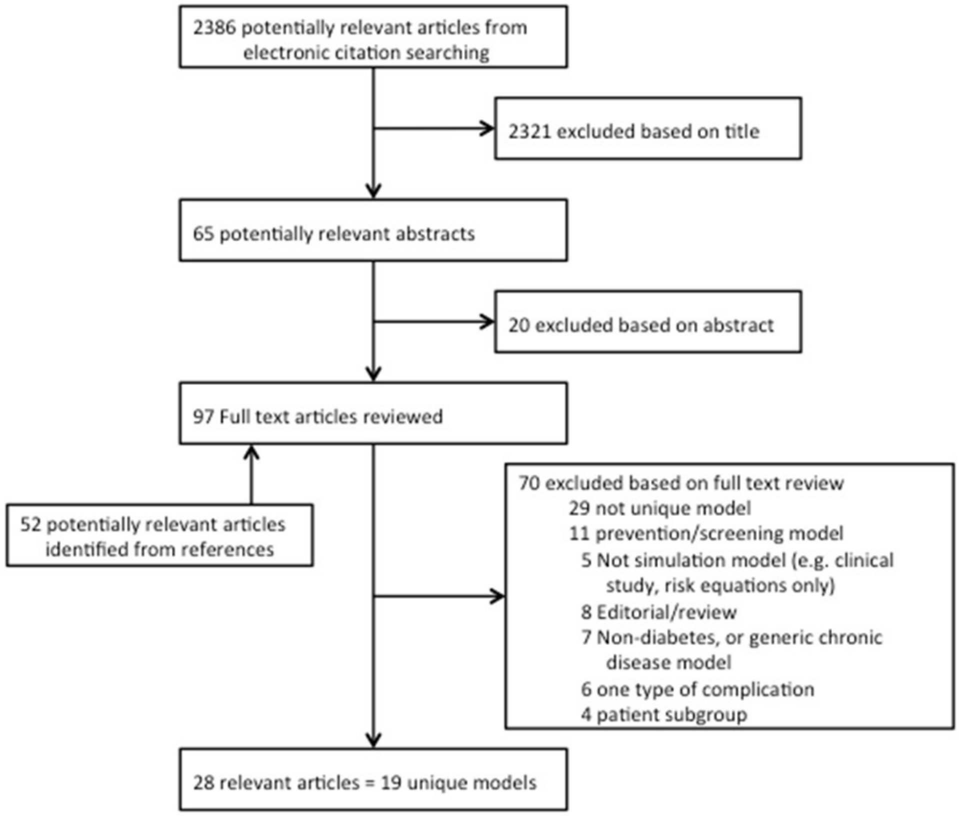

**Study Characteristics, Summary of Studies**

Table 1 provides a description of the models included in the review. As per the inclusion criteria, the models main aims were to evaluate therapies and interventions by predicting future medical events over a patient's lifetime. Twelve models investigated type 2 diabetes[23-34], two developed type 1 models[35, 36], two created separate models for type 1 and type 2[37, 38], and three developed joint type 1 and type 2 diabetes models.[39-41] The majority of models were developed either in the United States (9 models) or the United Kingdom (5 models), with the others developed elsewhere in Europe or Canada. The majority of later models use either data or methods from earlier models for development or validation.

**Table 1.** Summary of Characteristics of Included Models

| | Where? | Model Aims | Type of Diabetes | Model Type | Monte Carlo | Events/ Complications | Data Sources |
|---|---|---|---|---|---|---|---|
| **DCCT 1996**[35] | US | Estimate and compare benefits of intensive therapy of blood glucose compared to conventional therapy | T1 | Markov-based Monte Carlo microsimulation model similar to that developed by Javitt89 (Ophthalmology) | Yes | retinopathy (5 states), nephropathy (4 states), neuropathy (3 states), and mortality | DCCT<br>Large clinical trials and epidemiological studies |
| **Eastman 1997**[29, 46] | US | Predicts rates of microvascular complications, CVD and mortality to evaluate preventative interventions | T2 | Markov- based Monte Carlo microsimulation model similar to DCCT 1996 and Javitt89 (Ophthalmology) | Yes, random numbers generated to determine events | retinopathy (5 states), nephropathy (4 states), neuropathy (3 states), CVD morbidity (2 states), and mortality | Large clinical trials and epidemiological studies. Framingham used for CVD risk |
| **Vijan 1997**[33] | US | Calculates risks for developing blindness and renal disease for patients at different ages of diabetes onset and levels of glycaemic control | T2 | Markov-based cohort model | No | retinopathy (5 states), nephropathy (5 states), includes no comps and death | DCCT data, and cohort data including Rochester and WESDR. UKPDS used for external validation |
| **Brown 2000 (GDM)**[24] | US, UK, | Predicts medical futures of both individuals and populations with diabetes | T2 | Discrete-time microsimulation using continuous prediction equations | Yes, random numbers generated to determine events | retinopathy (5 states), nephropathy (4 states), neuropathy (3 states), 12 major-diabetes related CVD events and secondary events, all cause mortality, and ESRD mortality | Uses various data sources and literature to generate data and estimate events rates/ probabilities: Framingham, Kaiser Permente Northwest, UKPDS, NHANES III, US Renal data System |

**Table 1.** Summary of characteristics of included models (continued)

| | Where? | Model Aims | Type of Diabetes | Model Type | Monte Carlo | Events/Complications | Data Sources |
|---|---|---|---|---|---|---|---|
| **Palmer 2000 (Accuism)** [38, 45] | Swiss | Estimates medical and cost outcomes for progression of diabetes and related complications for different intervention strategies | T1, T2 | Markov-based cohort simulation model | No | retinopathy (4 states), nephropathy (9 states), acute MI (7 states), stroke (4 states), amputation (4 states), hypoglycaemia (3 states), ketoacidosis (3 states), lactic acidosis (T2 only), and complication specific and non-specific mortality | DCCT, UKPDS, Framingham |
| **Caro 2000**[25] | US | To assess the benefits and costs of troglitazone to improve glycaemic control in terms of long-term outcomes associated with diabetes | T2 | Markov-based Monte Carlo microsimulation model, modifies Eastman model to fit clinical trial circumstances | Yes | retinopathy (5 states), nephropathy (4 states), neuropathy (5 states), hypoglycaemia, macrovascular disease, and mortality | Trials and large epidemiological studies, UKPDS, DCCT, Rochester, Wisconsin data, Framingham risk |
| **Bagust 2001 (DiDCT)**[23] | UK | Estimate lifetime diabetes-related morbidities, and use the model for evaluation of policies and interventions in the treatment of diabetes | T2 | Markov-based cohort model, extensive redesign of the Eastman model | No | modules for diabetic therapy (4 broad classes), HbA1c profile, retinopathy (9 states), nephropathy (4 states), neuropathy (7 states), cardiovascular morbidity and different mortality risks for each complication, and estimates the prevalence of all CVD at all ages. A further 4 modules included (cataract removal, cancer prevalence, major hypoglycaemic events and multiple morbidity) | UKPDS, WESDR |

**Table 1.** Summary of Characteristics of Included Models (continued)

| | Where? | Model Aims | Type of Diabetes | Model Type | Monte Carlo | Events/Complications | Data Sources |
|---|---|---|---|---|---|---|---|
| **CDC 2002**[21, 26] | US | Estimated incremental cost-effectiveness of intensive glycaemic control, intensified hypertension control, and reduction in serum cholesterol | T2 | Markov- based cohort model building on Eastman and a screening model previously developed by CDC. [ref CDC screening] | Unclear, no mention of MC but Eastman and CDC screening was | retinopathy (3 states), nephropathy (5 states), neuropathy (6 states), coronary heart disease (6 states), stroke (4 states) and mortality | UKPDS, NHANES III |
| **Eddy 2003 (Archimedes)** [39, 51, 52] | US | A mathematical model of the anatomy, pathophysiology, tests, treatments and outcomes of diabetes that simulates what happens in real life in a real health care system at realistic and natural level of detail | Joint T1 and T2 | Continuous time model using differential equations and object-orientated programming. | No | Simulates: patients, anatomy and physiology, healthcare facilities and personnel, tests and treatments, outcomes. Model can be reduced in detail depending on the application | DCCT, UKPDS |
| **Clarke 2004 (UKPDS OM1)**[28] | UK | System of equations to estimate likely occurrence and timing of major diabetes and cardiovascular complications and death | T2 | Discrete-time Monte Carlo microsimulation | Yes, random numbers generated to determine events | First complications for: fatal or non-fatal MI, other IHD, stroke, congestive heart failure, amputation, renal failure, eye disease (blindness in 1 eye), death (immediate death, diabetes-related death and other death) | UKPDS |

**Table 1.** Summary of Characteristics of Included Models (continued)

| | Where? | Model Aims | Type of Diabetes | Model Type | Monte Carlo | Events/ Complications | Data Sources |
|---|---|---|---|---|---|---|---|
| **Palmer 2004 (CORE)** [41, 53] | Swiss, US | To determine long-term health outcomes and economic consequences of implementing different treatments for diabetes | Joint T1 and T2 | Markov-based Monte Carlo microsimulation model | Yes, random numbers generated to determine events | retinopathy (4 states plus screening, diagnosis, and treatment), nephropathy (7 states), neuropathy (2 states), foot ulcer (9 states), macular oedema (3 states), cataract (3 states), hypo (2 states), ketoacidosis (2 states), lactic acidosis (2 states), MI (3 states), angina (2 states), CHF (3 states), stroke (3 states), PVD (2 states), non-specific mortality | Framingham, UKPDS, DCCT, WESDR, EURODIAB and other published sources |
| **Mueller 2006 (EAGLE)** [40] | France, Germany, Spain, US, UK | To simulate diabetes-related complications and their impact on costs across a wide spectrum of different patient populations | Joint T1 and T2 | Discrete-time Monte Carlo microsimulation<br><br>NB: paper says discrete event, but it is discrete time since yearly increments | Yes | Retinopathy (6 states), nephropathy (3 states), neuropathy (4 states), hypoglycaemia, cardiovascular system (non-fatal MI, non-fatal angina pectoris, non-fatal heart failure, non-fatal stroke), specific cause mortality (fatal MI, fatal HF, fatal stroke, death after renal disease), all-cause mortality | DCCT, UKPDS, WESDR |

**Table 1.** Summary of Characteristics of Included Models (continued)

| | Where? | Model Aims | Type of Diabetes | Model Type | Monte Carlo | Events/ Complications | Data Sources |
|---|---|---|---|---|---|---|---|
| **Grima 2007**[37] | Canada | T1: determine health outcomes and consequences of different combinations of diabetes interventions in newly diagnosed T1<br>T2: Examine long term cost-effectiveness of insulin glargine compared to NPH insulin in patients with HbA1c>-7% | T1, T2 | Markov-based cohort model | No | 5 states overall relating to no complications, first non-fatal complication, second non-fatal complication, fatal complication, and death from other causes.<br>Complications for T1: hypoglycaemia, ketoacidosis, MI, stroke, amputation, nephropathy, retinopathy<br>Complications for T2: ESRD, amputation, retinopathy, MI, HF, stroke | UKPDS, Palmer00 |
| **Tilden 2007**[32] | UK | Compare costs and benefits of pioglitazone vs. rosiglitazone combined with metformin | T2 | Markov-based Monte Carlo cohort simulation model using UKPDS risk equations | Yes, assume random numbers generated to determine events | 8 states overall relating to combination therapy with or without history of complications, insulin therapy with or without history of complications, discontinued therapy therapy with or without history of complications, diabetes related death and non-diabetes related death. Events include IHD, blindness, non-fatal/fatal MI, non-fatal/fatal stroke, amputation, renal failure | UKPDS, RCT studies |

**Table 1.** Summary of Characteristics of Included Models (continued)

| | Where? | Model Aims | Type of Diabetes | Model Type | Monte Carlo | Events/ Complications | Data Sources |
|---|---|---|---|---|---|---|---|
| **Chen 2008 (JADE)**[27, 54] | US | Investigate the impact of alternative HbA1c thresholds for treatment intensification | T2 | Discrete-time Monte Carlo microsimulation with risk equations for each complication and death<br><br>NB: paper says discrete event, but it is discrete time since yearly increments | Yes, random numbers generated to determine events | First complications for: fatal or non-fatal MI, other IHD, stroke, congestive heart failure, amputation, renal failure, eye disease (blindness in 1 eye), death (immediate death, diabetes-related death and other death), and a treatment module which is updated based on HbA1c | UKPDS OM event equations, Real-life Effectiveness and Care Patterns of Diabetes Management study |
| **Willis 2013 (ECHO)** [34] | Sweden | To predict costs and health outcomes and to estimate the cost-effectiveness of alternative diabetes treatments | T2 | Markov-based microsimulation model | Yes, transition probabilities depend on time-dependent patient-level covariates from probabilistic distributions | Marco-vascular complications include IHD, MI, CHF, Stroke. Markov health states include micro-vascular complications [retinopathy (10 states), nephropathy (4 states), neuropathy (7 states)], and adverse events of treatment [Hypo (2 states), Other (4 states)]. 3 mortality equations. | UKPDS for macrovascular events and mortality equations, transition probabilities from NIH and DiDACT model and structure from CORE |

**Table 1.** Summary of characteristics of included models (continued)

| | Where? | Model Aims | Type of diabetes | Model Type | Monte Carlo | Events/ Complications | Data Sources |
|---|---|---|---|---|---|---|---|
| **Hayes 2013 (UKPDS OM2)**[30] | UK | Simulate diabetes-related complications over a lifetime and calculate health outcomes by re-estimating the original risk and mortality equations, estimating equations for new events, and the use of new risk factors | T2 | Discrete-time Monte Carlo microsimulation with risk equations for each complication and death | Yes, random numbers generated to determine events | First complications: fatal or non-fatal MI, other IHD, stroke, congestive heart failure, amputation, renal failure, eye disease (blindness in 1 eye), diabetic ulcer of the lower limb; secondary complications: MI, stroke and amputation; death (immediate death, diabetes-related death and other death) | UKPDS, Lipids in Diabetes Study (LDS) |
| **Kruger 2013 (Sheffield)**[36, 55] | UK | Allow a number of cost-effectiveness evaluations for adults with type 1 diabetes | T1 | Markov-based microsimulation model | Unclear, although random numbers generated to determine events and can be used to perform probabilistic sensitivity analysis | retinopathy (5 states), nephropathy (5 states, including death from ESRD), neuropathy (3 states), cardiovascular (angina, MI, stroke, heart failure, death from stroke, MI and HF), acute complications (hypoglycaemia, DKA), other mortality | DCCT, WESDR, UKPDS, Eastman, EDIC (Epidemiology of Diabetes Interventions and Complications) |

There are four main types of models that were reviewed: five were Markov-based cohort models[23,26,32,33,37,38]; six were Markov-based microsimulations[25,29,34-36,41]; five were discrete-time microsimulations using risk equations to determine events[24,27,28,30,40]; two models were a combination of Markov and discrete-time risk equations[31,32]; and one model used continuous time differential equations.[39] A discrete-time simulation, also known as time-slicing, models the progression through time in which a constant time step is adopted.[42] The model then assesses which events have happened at the end of each time step (or cycle). Multiple events can occur in each cycle until death. Markov-type models also, typically, use discrete-time steps.[43] Markov models are state-transition models that assess the probabilities of transition to determine if a patient has moved from one state to another at the end of each cycle. The main assumption around Markov models is that it is memoryless. In other words, the transition to another state relies solely on the current state. However, techniques have been developed to mimic memory in these types of models.[44] Markov models can be analysed using a cohort of patients, or following the path of an individual.

Of the models, eleven used Monte Carlo techniques[24,25,27-30,32,34,35,40,41], five did not[23,33,37,39,45], and three were unclear in their descriptions[26,31,36]. Monte Carlo simulation is used in order to provide a more stable estimate of outcomes or probabilistic answers to the simulation.

**ADA Guidelines**

Table 2 provides a coding system for adequacy of reporting of the ADA criteria. In order for models to be reproducible, transparency is a key component of reporting a model. When assessing the transparency of the reporting of the models, we determined if a model diagram was reported along with equations, transition probabilities or other information relating to determination of events or transition between states. Nine models[24,27-30,33,34,36,41] provided a model diagram and a description of further details needed to determine events or transitions, thereby providing adequate transparency in the model. A further six models[23,26,32,35,37,45] provide a model diagram in detail but not all the details required to determine events/transitions; two models[31,39] provide diagrams for some events but not a full diagram; and two models[25,40] did not provide a model diagram in the paper.

Internal validation of models is reported in over half of the models reviewed, either within the original paper or as a companion article. External validation was less frequently reported alongside the original descriptive paper, though many have been validated in follow-up articles years after first publication.

ADA guidelines suggest different types of uncertainty that can be assessed, and suggestions on how to assess them. They advise assessing ignorance and known variability through sensitivity analysis, of which all models reported in some way. The majority of models reported both performing sensitivity and providing results within the article, with only five exceptions: one model[24] was unclear in their descriptions of sensitivity analysis by stating it could be performed but did not detail what variables or parameters could be varied or report the results; two models state that sensitivity analysis was performed but did not report the results[23,40]; one did not mention sensitivity analysis[28]; and one model[39] reported that uncertainty was dealt with through extensive validation, making it unclear how uncertainty was dealt with. Statistical variability (parameters derived from statistical analysis) was less well reported. ADA suggests either reporting confidence intervals for the parameter, confidence intervals for model results that depend on that parameter, or sensitivity analysis. However, for most models confidence intervals were not reported for the parameter, or sensitivity analyses performed around the parameters. Over half of the models reported the use of Monte Carlo techniques for dealing with uncertainty.

**Table 2.** Criteria Defined by the ADA in Reporting a Diabetes Prediction Model

| | Transparency | Validation | | Uncertainty | | | Diabetes-specific Requirements | | | | | |
|---|---|---|---|---|---|---|---|---|---|---|---|---|
| | | Internal | External | Ignorance and known variability | Statistical variability | 1st order Monte Carlo | Long-term | Competing risks | Interdependence | Range of events | Length and quality of life | Perspective |
| DCCT 1996 | + | - | - | ++ | - | ++ | ++ | - | - | - | ++ | ++ |
| Eastman 1997 | ++ | ? | - | ++ | - | ++ | ++ | - | - | + | + | ++ |
| Vijan 1997 | ++ | ++ | ++ | ++ | ++ | - | ++ | + | - | - | - | - |
| Brown 2000 (GDM) | ++ | - | - | ? | - | ++ | ++ | ++ | - | ++ | ++ | ++ |
| Palmer 2000 (Accuism) | + | - | ++ | ++ | ++ | - | ++ | ++ | ? | ++ | + | ++ |
| Caro 2000 | - | ? | - | ++ | ? | ++ | ++ | - | - | + | ++ | ++ |
| Bagust 2001 (DiDCT) | + | ? | - | + | ? | - | ++ | + | ? | + | ? | ++ |
| CDC 2002 | + | ++ | ++ | ++ | - | ? | ++ | ++ | ? | + | ++ | ++ |
| Eddy 2003 (Archimedes) | + | ++ | ++ | ? | ? | - | ? | + | ++ | ++ | ? | ? |
| Clarke 2004 (UKPDS OM1) | ++ | ++ | - | - | ++ | ++ | ++ | ++ | ++ | ++ | ++ | ? |
| Palmer 2004 (CORE) | ++ | ++ | ++ | ++ | ? | ++ | ++ | ++ | ++ | ++ | ++ | ++ |
| **Publication of ADA guidelines** | | | | | | | | | | | | |
| Mueller 2006 (EAGLE) | + | ++ | - | + | ? | ++ | ? | ++ | - | ++ | + | - |
| McEwan 2006 (Cardiff/DiabForecaster) | + | ++ | - | ++ | - | ? | + | ? | ? | + | + | - |
| Grima 2007 | + | ++ | + | ++ | + | - | + | ++ | - | + | ++ | ++ |
| Tilden 2007 | + | - | - | ++ | + | ++ | ++ | ++ | ++ | ++ | ++ | ++ |
| Chen 2008 (JADE) | ++ | + | + | ++ | + | ++ | ++ | ++ | ++ | ++ | ++ | - |
| Willis 2013 (ECHO) | ++ | ++ | ++ | ++ | + | ++ | ? | ++ | ? | ++ | ++ | - |
| Hayes 2013 (UKPDS OM2) | ++ | ++ | - | ++ | ++ | ++ | ++ | ++ | ++ | ++ | ++ | - |

++ reported well; + partially reported; ? unclear reporting; - not reported

The inclusion criteria determined that all models would be lifetime models. However, some do not report for how long the model is run, or cap the model at a particular age. For example, the Cardiff model stops after 20 years, but can be extended to 40 years if further information is provided to the model.[31] Other models do not provide an estimate of the end point for the model, or for how long it can conceivably run.[34,39,40] In early models, competing risks were not well reported or incorporated. However, only three models did not report any incorporation of competing risks.[25,29,35]

Interdependence is the least well reported of the diabetes specific criteria. The Archimedes model in 2003 and UKPDS in 2004 were the first models to explicitly report that interdependence was modelled between the complications. Neither of these models were Markov-based models, where interdependence may be difficult to incorporate between the different states. However, most Markov-based microsimulation models after this also included interdependence between types of events in some way, such as tracker variables. UKPDS, being the first discrete-time microsimulation, incorporated interdependence through the use of indicator variables. Consequently, many later models, such as the JADE model and ECHO amongst others, incorporated event equations from the UKPDS model, and so incorporated interdependence.

All models include retinopathy or blindness, and nephrology, renal failure or end-stage renal disease (ESRD) as possible complication events. All but one model includes neuropathy or amputation.[33] Eastman[29] and GDM[24] include a CVD module where states included yes or no but Accuism[45] was the first to incorporate specific CVD complications as modules or events in the model. Following this, all models included both micro- and macrovascular complications.

Length of life is reported in all but one of the models, which instead reported duration of treatment to prevent 1 year of blindness.[33] One model did not report QALY[45]; two studies were unclear in their reporting if QALYs or QALEs could be calculated using the model[23,39]; and one reported QALYs in a companion paper.[46] Perspective of costs was not often reported, mainly because the paper reports the model description and method and costs, QALYS and perspective are reported in application papers published after the initial article.

### Discussion

This article aimed to critically appraise how well the models are described in relation to the criteria described by the ADA guidelines, which included four main areas: transparency, validation, uncertainty and diabetes specific criteria. In total, 19 models were identified which were described in over 28 articles. The majority of models provided adequate descriptions of the models, although a clear, descriptive short summary of the model was often lacking. In early diabetes modelling, before the ADA guidelines were published, the majority of models did not include descriptions of all the diabetes specific components of the ADA guidelines. However, by 2004 most models were reporting these components. All models were long-term diabetes models with the majority providing estimates of length and quality of life, and incorporating a wide range of compilations from various organ systems. However, descriptions of model validation and uncertainty were the most poorly reported of the four main areas, with interdependence between the complications being the least well incorporated or reported of the diabetes-specific criterion. These areas will be discussed further.

### Validation

Model transparency does not indicate that a model is accurate. Therefore, it is important that a model is also valid. Validation aims to determine if a model accurately calculates the outcomes of interest.[10] Five main types of validation are identified: face validity, verification (internal validity), cross validity, external validity and predictive validity. The ADA guidelines recommend describing internal validity and external validity of a model.

Internal validity is the extent to which a model reproduces the results of the studies that are used to develop the model.[17] In other words, ensuring that the model behaves as intended and has been implemented correctly.[10] The ISPOR-SMDM Modelling Good Research Practices Task Force recommend that all model should be subjected to rigorous verification and the methods used should be described in the non-technical documentation of the model. The majority of models in this review report internal validation either within the original paper or as a companion paper. The authors often only state that the model was validated against the data from which it was created but do not provide specific details. However, this does not mean that further details are not available elsewhere, such as a non-peer reviewed report or requested from authors.

External validation should show if the model can reproduce results of studies that were not used to develop the model and involves three steps: identifying data sources, running the model and comparing the results. Models often draw on the same data sources in order to populate or create the models. For example, the UKPDS dataset[47] and risk equations[28] are used for creation by all but three models, two of which were published before the UKPDS trial[29,35], and one that used in for external validation.[33] This poses a problem for externally validating the models. Due to the lack of appropriate, independent data at the time of model creation, external validation often occurs years after the model was first reported, for example the CDC model.[48] The UKPDS trial was one of very few large trials conducted that could be used for validation, but if the majority of the other models already use the UKPDS risk equations in their construction then this would exclude it from being used for independent external validation. Another complication in the effort to externally validate a model occurs when older datasets are not necessarily relevant today given new drugs on market, lifestyle interventions and better standard of living/life expectancy. Additionally, validation of a model using one data source does not necessarily make it valid, as it would need to be tested using various patient groups, timescales and other factors.[10,49] However, the Mount Hood diabetes challenge (http://www.mthooddiabeteschallenge.org/) has been instrumental in encouraging external validation (and cross validation) by issuing 'short term' challenges designed to look at how models predict trials that have been published after model development. The Mount Hood Diabetes Challenge provides a platform to externally validate models. The conference brings together diabetes researchers to help solve the challenge of treating diabetes, discuss the modelling of diabetes progression and diabetes complications. Hypothetical modelling challenges are provided to modelling groups in advance of the conference, and the results are compared and examined during the conference.

#### Uncertainty

Uncertainty analysis aims to assess the confidence in a chosen course of action and determine the value of additional information to inform a decision.[11] All models included in the review assessed uncertainty through some means, either by deterministic sensitivity analysis, probabilistic sensitivity analysis or Monte Carlo simulations. However, Expected Value of Perfect Information (EVPI) and Expected Value of Perfect Parameter Information (EVPPI) were not assessed or reported by any of the models. The Archimedes model[39] aimed to assess uncertainty through extensive validation, but the authors concede that this approach introduces further error and bias since trials are subject to random and systematic errors.

The structure of the model will determine whether Monte Carlo error (of stochastic error) can be ignored.[11] For individual patient simulation, such as the discrete-time models or Markov-based microsimulations, Monte Carlo error needs to be eliminated before addressing parameter uncertainty. Of the models included in this review, five were Markov-based cohort models, which do not require elimination of Monte Carlo error. This means that the model articles did not report the use of Monte Carlo techniques, as it was not required to assess uncertainty.

Bagust et al[23] argues against the use of microsimulation and Monte Carlo simulation. They argue that in long-term models using these techniques introduces further uncertainty in the model due to the assumptions around the source and nature of variations. Also, the authors believe that any confidence regions obtained will offer little information due to being too wide. The authors suggest that the only practical approach to assessing uncertainty is to perform selective sensitivity analysis. However, recent guidelines[11] suggest that selectively varying the inputs of the model to assess the changes on the outputs should only be used as a measure of sensitivity and does not represent uncertainty in the parameters.

#### Interdependence

Previous to 2003 (and therefore the publication of the ADA guidelines) many models did not include adequate descriptions of the diabetes specific requirements as outlined by ADA. Interdependence between diabetes endpoints was least well reported or incorporated by the models. However, the type of model used may be a possible explanation for this. Twelve models were Markov based models (six microsimulation), five were discrete-time, one was a combination of discrete-time and Markov-based modelling, and the last used continuous time differential equations. The different modelling approaches have different strengths and limitations.

Cohort Markov models simplify the model into a discrete number of states and minimise computing time by simulating a cohort and not individual patients. Markov microsimulation models allow variation between individual patients to be modelled. Markov models by definition do not carry a history of events or of time spent in previous states. This can be overcome by incorporating temporary or tunnel states, which can only be visited in a specific sequence[44]; or tracker variables, which update when an event has taken place.[43] Tracker variables will generally only be incorporated when the model is a microsimulation meaning that a record of that patient's movements may be recorded.

Discrete-time models allow a larger number of possible events to be included and can more easily incorporate interdependence between types of events. This was achieved by including an indicator variable that would update when an event occurred. Three discrete-time models were described as discrete-event models within the article. However, each of these models actually used a discrete-time step in the simulation. A discrete-time simulation, also known as time-slicing, models the progression through time in which a constant time step is adopted.[42] The model then assesses which events have happened at the end of each time step. In contrast, in a discrete-event simulation, the simulator need not explicitly represent the state of the system at non-event times and can therefore move from one event to the next without simulating all time-steps in between.[9,50] None of the models included in this review utilise a discrete-event simulation structure.

Continuous-time differential equations have many advantages, including preserving the continuous nature of risk factors, and incorporating this into interactions between comorbidities, complications and disease. Thus interdependence is achieved between all disease, complications and comorbidities included in the model. However, due to the complex nature of the more advanced mathematics used, the model is less transparent due to fewer people having the training and knowledge to use these models.[39]

### CONCLUSIONS

After the publication of the ADA guidelines, several models reference the guidelines and work towards better reporting based on them. The guidelines themselves do not take into account that some requirements are unnecessary for inclusion in the model. For example, Markov-based cohort models do not require the elimination of Monte Carlo error and so this will not be reported. Bagust and McEwan[49] warn that strictly following the guidelines may mislead users into believing that the models are accurate and reliable. They state that models are only a tool to guide decision makers and not objective evidence.

This is the first review to assess diabetes model on how adequately published diabetes models report on the criteria set out by the ADA guidelines for diabetes modelling. Diabetes modelling is still in its relative infancy with the first major model published in 1996. As more models have been developed, the quality of reporting of the model has improved, but more emphasis should be placed on including a clear, descriptive short summary of the model; reporting the validation procedures; the assessment of uncertainty in models; and incorporating interdependence between complications.

**Declaration of Competing Interests**

The authors confirm that this is original work that has not been published or submitted for publication elsewhere, and that there are no conflicts of interest for any author. Author contributions are as follows. LG designed and carried out the systematic search and drafted the manuscript. All authors were involved in design of the study, decision for inclusion and exclusion of articles, and editing of the manuscript for submission. All authors read and approved the final manuscript.
